# Sjögren’s disease and concomitant fibromyalgia: clinical profile and implications for disease activity assessment

**DOI:** 10.1007/s11739-025-04193-x

**Published:** 2025-11-11

**Authors:** Stefano Stano, Vincenzo Venerito, Daniele Domanico, Maria Iacovantuono, Eduardo Urgesi, Fabio Cacciapaglia, Maria Giannotta, Marco Fornaro, Paola Conigliaro, Antonio Vitale, Maria Sole Chimenti, Florenzo Iannone, Giuseppe Lopalco

**Affiliations:** 1 Rheumatology Unit - Department of Precision and Regenerative Medicine, Jonian Area (DiPReMeJ), University of Bari “Aldo Moro”, Piazza G. Cesare 11, 70124 Bari, Italy; 2 Rheumatology, Allergology and Clinical Immunology, Department of Systems’ Medicine, University of Rome Tor Vergata, Rome, Italy; 3 Department of Medicine, University Cardiologic Unit - Interdisciplinary, Policlinico University Hospital, University of Bari “Aldo Moro”, Bari, Italy; 4 Department of Medicine and Surgery, Rheumatology Unit, “F. Miulli” General Hospital, LUM University ‘G. De Gennaro’, Casamassima, Bari, Italy; 5 Department of Medical Sciences, Surgery and Neurosciences, Research Center of Systemic Autoinflammatory Diseases and Behçet’s Disease Clinic, University of Siena, Siena, Italy

**Keywords:** Sjögren’s disease, Fibromyalgia, Overlap, Disease activity, Precision medicine

## Abstract

Fibromyalgia (FM) is more prevalent in patients with Sjögren’s disease (SjD) than in the general population and a bidirectional association between the two conditions has been proposed. However, the clinical profile of patients with concomitant FM and SjD remains poorly characterized. This study aimed to assess the prevalence and clinical correlates of FM in a multicenter Italian cohort of patients with SjD. Patients fulfilling the 2016 ACR–EULAR classification criteria for SjD were retrospectively evaluated. FM was defined according to the 2016 diagnostic criteria. Clinical, serological, and therapeutic data were compared between patients with and without FM. Logistic regression models identified factors associated with FM. Among 267 patients with SjD (95% female, median age 60), FM was diagnosed in 30%. Patients with FM reported significantly higher symptom burden, as measured by the EULAR Sjögren’s Syndrome Patient-Reported Index (median 7.7 vs. 6.0; *p *< 0.001), with all individual domains, namely pain, fatigue, and dryness, being significantly increased (*p* < 0.01, for all). In contrast, EULAR Sjögren’s Syndrome Disease Activity Index scores were comparable between groups (*p* = 0.808). In the logistic regression model, three variables were independently associated with FM: higher symptom burden (adjusted odds ratio (aOR 1.36, 95% CI 1.13–1.62; *p* = 0.001), mixed anxiety–depressive disorder (aOR 3.24, 95% CI 1.13–9.30; *p* = 0.029), and corticosteroid use (aOR 2.76, 95% CI 1.02–7.48; *p* = 0.046). In patients with SjD, FM is associated with a higher symptom burden despite similar disease activity level. These findings highlight the need to distinguish symptom amplification from true inflammatory activity, limiting unnecessary corticosteroid use.

**Supplementary Information:**

The online version contains supplementary material available at 10.1007/s11739-025-04193-x.

## Introduction

Sjögren’s disease (SjD) is a systemic autoimmune disorder characterized by chronic epithelitis due to lymphocytic infiltration of exocrine glands, most commonly resulting in xerostomia and xeropthalmia. Less frequently, SjD may affect additional exocrine glands and involve various internal organs [1]. In addition to sicca symptoms, patients often experience chronic fatigue and widespread pain, which are frequently disabling and significantly impair quality of life [2].

The pathogenesis of SjD is not fully understood, although B-cell hyperactivation, immune complex formation, and autoantibody production play central roles [3]. In Italy, the prevalence of SjD is estimated at 3.8 per 10,000 inhabitants, with a mean age at diagnosis of approximately 60 years and a striking female-to-male ratio of 15:1 [4]. A recent Italian study demonstrated that SjD patients have substantially greater pharmacological, inpatient, and outpatient healthcare needs, resulting in healthcare costs that are three times higher than those of the general population. [4] Consistently, their quality of life is significantly impaired and comparable to that of patients with systemic lupus erythematosus (SLE) [5].

A Brazilian study recently identified pain and fatigue as the main predictors of poor quality of life in SjD, regardless of age or disease activity [6]. Pain in SjD can arise from both nociceptive and neuropathic mechanisms, and pain-related syndromes, notably fibromyalgia (FM), have been frequently reported [7].

FM is a chronic pain syndrome marked by widespread pain, fatigue, sleep disturbances, and idiopathic somatic symptoms [8]. Its complex clinical presentation often includes headaches, dryness, autonomic dysfunction, cognitive disturbances, hypersensitivity to external stimuli, and psychiatric disorders [9]. FM affects approximately 2–3% of the global population, with a female/male ratio of 4:1, and has an even higher prevalence among patients with chronic diseases such as type 2 diabetes mellitus (T2DM), SLE, spondylarthritis (SpA), and Behçet’s disease (BD) [10]. In Italy, the estimated prevalence of FM is 3.7%, with similar sex distribution [11].

Although the exact pathogenesis of FM remains elusive, three major mechanisms have been proposed: central sensitization (linked to alterations in neurotransmitter regulation), peripheral sensitization (involving hyperactive nociceptors), and immune/inflammatory pathways. Furthermore, genetic and endocrine factors may contribute to disease susceptibility [12].

A recent meta-analysis reported that FM is more frequently diagnosed in patients with autoimmune diseases, including rheumatoid arthritis (RA), axial spondyloarthritis (SpA), and psoriatic arthritis (PsA), with pooled prevalence estimates of 18–24% in RA, 15% in axial SpA, and 18% in PsA [13]. The link between FM and autoimmune diseases has been further supported by Sedda et al., who demonstrated shared pro-inflammatory cytokines and neurochemical mediators as potential mechanisms underlying this overlap [14].

Moreover, a bidirectional association between SjD and FM has also been reported [15]. Notably, a nationwide Taiwanese study found that FM patients have an increased risk of developing SjD, particularly between the ages of 20 and 49 years [16]. However, data on the prevalence and clinical characteristics of FM in Italian patients with SjD remain limited.

This study aimed to investigate the current prevalence and clinical profile of FM in an Italian multicenter cohort of patients with SjD, and to compare the clinical and serological characteristics of SjD patients with and without FM.

## Methods

### Study population and data collection

Consecutive patients visited at least twice from January 2017 to January 2025 in our third-level rheumatology outpatient clinic, with a diagnosis of SjD fulfilling the 2016 ACR–EULAR classification criteria [17]—with or without FM meeting the updated 2016 FM diagnostic criteria [18]—were included in the study. The diagnosis of FM was confirmed in all SjD patients by the visiting rheumatologist during the outpatient clinical assessment. Demographic, clinical, laboratory, and instrumental data were retrospectively collected from medical records and compared between patients with and without associated FM.

Clinical data, including the presence of specific symptoms or organ involvement attributable to SjD, were reported. Ocular dryness was assessed using the Schirmer test, while salivary gland biopsy was performed to confirm the diagnosis of SjD in seronegative patients or if clinically required. The presence of interstitial lung disease (ILD) was confirmed by high-resolution computed tomography (HRCT) scan of the lungs in patients presenting with suggestive respiratory symptoms or spirometry abnormalities, including reduced forced vital capacity (FVC) or diffusing capacity for carbon monoxide (DLCO). Data on comorbidities such as osteoporosis, T2DM, and mixed anxiety–depressive disorder (MADD) were also collected. Laboratory parameters included: complete blood count, serum protein electrophoresis, cryoglobulins, β2-microglobulin, immunoglobulins classes (IgA, IgG, IgM), erythrocyte sedimentation rate (ESR), C-reactive protein (CRP), lactate dehydrogenase (LDH), and complement components C3 and C4. Metabolic parameters assessed the lipid profile (total cholesterol, high-density lipoprotein (HDL), low-density lipoprotein (LDL), triglycerides) and fasting morning blood glucose. Autoantibody assessment encompassed antinuclear antibodies (ANA), anti-Sjögren’s syndrome-related antigen A (SSA), anti-Sjögren’s syndrome-related antigen B (SSB), and rheumatoid factor (RF).

### Assessment of disease activity and treatments

Disease activity was evaluated using validated indices specific for SjD, including the European Alliance of Associations for Rheumatology (EULAR) SS Patient-Reported Index (ESSPRI), which captures patient-reported symptom severity (pain, fatigue, and dryness), and the EULAR Sjögren’s Syndrome Disease Activity Index (ESSDAI), which encompasses clinician-assessed systemic and serological domains [19].

Previous and ongoing therapies were recorded, including corticosteroids (CS), expressed as daily prednisone-equivalent (PDNeq) dose, conventional synthetic disease-modifying antirheumatic drugs (csDMARD), biologic DMARD (bDMARD), pilocarpine, muscle relaxants, and antidepressants.

The study was conducted in accordance with the ethical principles of the 1975 Helsinki’s Declaration and was approved by the local ethics committee (Ethics Review Board of the Bari Policlinico, protocol n° 5277). Written informed consent, including authorization for the processing of personal data, was obtained from all participants. The study adhered to the Strengthening the Reporting of Observational Studies in Epidemiology (STROBE) guidelines [20].

### Statistical analysis

Data were retrospectively collected from medical records and reported into a dedicated database. Continuous variables were reported as means with standard deviations (SD) or medians with interquartile ranges (IQR), as appropriate. Categorical variables were expressed as absolute numbers and percentages. The D’Agostino–Pearson test was used to assess the normality of distribution. Comparisons between groups were performed using the paired t-test or the Mann–Whitney U test for continuous variables, and the Chi-square test for categorical variables.

Univariate logistic regressions were first performed to evaluate the association between each variable of interest and the presence of FM. Variables demonstrating a statistically significant association (*p* < 0.20) were selected for inclusion in the multivariate logistic regression model as listed in Supplementary Table 1. A backward stepwise selection procedure was applied to identify independent associations while adjusting for potential confounders of interest. Results are reported as odds ratios (OR) with 95% confidence intervals (CI), OR range, or adjusted (a) OR, as appropriate. A two-tailed *p*-value < 0.05 was considered statistically significant. Missing data were handled using a complete-case approach without imputation. All statistical analyses were performed using StataMP (v. 18).

## Results

### Demographic, clinical, and disease activity features

A total of 267 SjD patients were included in the study, of whom 254 (95.1%) were female, with a median age of 60 years (IQR 51–70). A concomitant diagnosis of FM was identified in 80 patients (30%). Patients with FM tended to be younger and more often female, although these differences were not statistically significant. Patients with FM had a significantly longer disease duration (median 120 months, IQR 72–192; *p* = 0.046) and follow-up period (*p* = 0.034) compared to those without FM. The prevalence of osteoporosis and T2DM was comparable between groups, whereas MADD we significantly more frequent in patients with FM (20% vs. 6.9%; *p* = 0.002).

No significant differences were observed in other clinical manifestations, including dryness, salivary gland swelling, or arthritis. However, arthralgias were more commonly reported in patients with FM (55% vs. 40.6%; *p* = 0.031). The prevalence of neurological, kidneys, skin, and lymphoproliferative involvement did not differ significantly between groups (Table 1).

**Table 1:** Baseline demographics and clinical characteristics of patients with Sjögren’s disease with and without fibromyalgia

Demographics and clinical characteristics	SjD (*n*. 267)	SjD with FM (*n*. 80)	SjD without FM (*n*. 187)	*p*-value
Female, *n*. (%)	254 (95.13)	79 (98.75)	175 (93.58)	0.07
Age (years), median (IQR)	60 (51—70)	57 (51—67)	61 (52—70)	0.09
Disease duration (months), median (IQR)	120 (72—192)	132 (72—180)	118 (69—192)	**0.05**
Follow-up duration (months), median (IQR)	69 (24—178)	128 (31- 214)	62 (20—152)	**0.03**
Osteoporosis, *n*. (%)	88 (33.08)	22 (27.85)	66 (35.29)	0.24
T2DM, *n*. (%)	18 (6.95)	6 (7.59)	12 (6.67)	0.79
MADD, *n*. (%)	29 (10.86)	16 (20)	13 (6.95)	** < 0.01**
Dryness, *n*. (%)	246 (92.13)	76 (95)	170 (90.91)	0.25
Exocrine glands swelling, *n*. (%)	30 (11.24)	9 (11.25)	21 (11.23)	0.99
Arthritis, *n*. (%)	33 (12.36)	10 (12.5)	23 (12.3)	0.96
Arthralgia, *n*. (%)	120 (44.94)	44 (55)	76 (40.64)	**0.03**
CNS involvement, *n*. (%)	4 (1.50)	1 (1.25)	3 (1.60)	0.83
PNS involvement, *n*. (%)	34 (12.73)	13 (16.25)	21 (11.23)	0.26
Kidney involvement, *n*. (%)	11 (4.12)	3 (3.75)	8 (4.28)	0.84
Skin involvement, *n*. (%)	47 (17.6)	13 (16.25)	34 (18.18)	0.70
Lymphoma, *n*. (%)	9 (3.38)	3 (3.80)	8 (3.21)	0.81

*CNS* central nervous system, *T2DM* type 2 diabetes mellitus, *ESSDAI* EULAR Sjögren syndrome disease activity index, *ESSPRI* EULAR Sjögren syndrome patient-reported index, *FM* fibromyalgia, *IQR* interquartile range, *MADD* mixed anxiety–depressive disorder, *n* number, *PNS* peripheral nervous system, *SjD* Sjögren’s disease, *VAS* visual analog scale

**Fig. 1:**
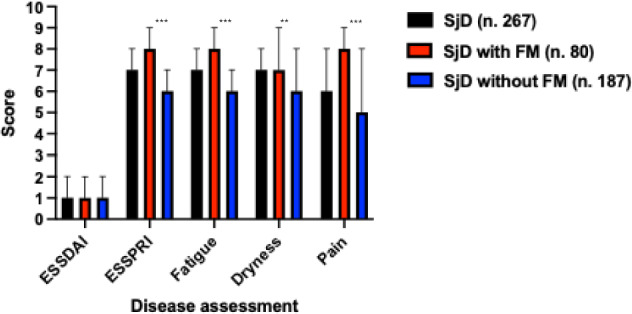
Comparison of the ESSDAI, ESSPRI, and ESSPRI domains (fatigue, dryness, pain) according to FM status in patients with SjD. Bars represent median and IQR. SjD with FM patients showed significantly higher ESSPRI, fatigue, and pain scores compared with SjD without FM (*p* < 0.01), while ESSDAI scores were comparable across groups. *ESSDAI* EULAR Sjögren’s syndrome disease activity index, *ESSPRI* EULAR Sjögren’s syndrome patient-reported index, *FM* fibromyalgia, *SjD* Sjögren’s disease, *=p<0.05, **=p<0.01, ***=p<0.001.

In terms of disease activity and patient-reported symptoms, ESSDAI scores were comparable between groups (median: 1 [IQR: 0–2]). On the contrary, patients with FM reported higher symptom burden as measured by ESSPRI (median: 7.7 vs. 6.0; *p* < 0.001). All three ESSPRI domains (fatigue, pain, and dryness) were significantly higher in the FM group (*p* < 0.001, *p* < 0.001, and *p* = 0.005, respectively), as shown in Fig. 1. Objective measures of glandular and pulmonary involvement showed no significant differences between groups, including Schirmer test (74% positive), salivary gland biopsy (50% positive), ILD on HRCT (< 10%), and median DLCO (75%) (Table 2).

**Table 2:** Disease activity scores and objective measures of glandular and pulmonary involvement

Disease assessment and organ involvement	SjD (*n*. 267)	SjD with FM (*n*. 80)	SjD without FM (*n*. 187)	*p*-value
ESSDAI, median (IQR)	1 (0—2)	1 (0—2)	1 (0—2)	0.59
ILD at HRCT scan, *n*. (%)	25 (9.36)	10 (12.5)	15 (8.02)	0.25
FVC (%) last, median (IQR)	107 (96—118)	110 (105—118)	106 (94—118)	0.19
DLCO (%) last, median (IQR)	75 (63—90)	74 (68—87)	76 (63—96)	0.79
Schirmer test positive, n. (%)	142 (73.58)	46 (73.02)	96 (73.58)	0.90
Focus score positive at biopsy, n. (%)	59 (49.58)	21 (55.26)	38 (46.91)	0.40
ESSPRI, median (IQR)	7 (5—8)	8 (7—9)	6 (4—7)	** < 0.001**
Fatigue VAS (0–10), median (IQR)	7 (5—8)	8 (7—10)	6 (3—8)	** < 0.001**
Dryness VAS (0–10), median (IQR)	7 (5—8)	7 (5—9)	6 (5—8)	** < 0.01**
Pain VAS (0–10), median (IQR)	6 (3—8)	8 (6—9)	5 (1—8)	** < 0.001**

*DLCO* diffusing capacity of carbon monoxide, *ESSDAI* EULAR Sjögren syndrome disease activity index, *ESSPRI* EULAR Sjögren syndrome patient-reported index, *FM* fibromyalgia, *FVC* forced vital capacity, *HRCT* high-resolution computed tomography, *ILD* interstitial lung disease, *IQR* interquartile range, *n*., number, *SjD* Sjögren’s disease, *VAS* visual analog scale

### Serological and laboratory findings

Inflammatory markers such as ESR and CRP were elevated in 32% and 13% of patients, with no significant differences between SjD patients with and without FM. White blood cell counts, including lymphocytes and neutrophils values, were within normal limits and similarly distributed between groups. Conversely, platelets counts were significantly higher in patients with FM (*p* = 0.002), although the frequency of thrombocytosis did not differ between groups. Elevated LDH (20%) and complement consumption (26%) were less frequent and showed no significant differences between groups. The lipid profile was within the normal range in most cases and comparable between groups. Hypergammaglobulinemia was detected in 35% of patients and was slightly more frequent in those without FM, though the difference was not statistically significant. Circulating cryoglobulins were found in less than 10% of our cohort, and β2-microglobulin levels remained within the normal range in all groups. Serum immunoglobulin levels were normal in both subgroups, with no significant differences. The autoantibodies profile was evenly distributed between groups. Briefly, ANA were positive in 79% of patients, anti-SSA in 70%, anti-SSB in 37%, and RF in 19% of the cases (Table 3).

**Table 3:** Serological and laboratory findings

Laboratory parameters	SjD (*n*. 267)	SjD with FM (*n*. 80)	SjD without FM (*n*. 187)	*p*-value
ESR increase (> 20 mg/L), n. (%)	86 (32.33)	23 (29.11)	63 (33.69)	0.47
CRP increase (> 5 mg/L), n. (%)	35 (13.16)	9 (11.39)	26 (13.90)	0.15
Leukocytes, N*10^9^/L, median (IQR)	5.35 (4.36—6.31)	5.49 (4.37—6.57)	5.33 (4.35—6.31)	0.82
Neutrophils, N*10^9^/L, median (IQR)	2.82 (2.30—3.85)	2.77 (2.20—3.7)	2.85 (2.33—3.9)	0.42
Lymphocytes, N*10^9^/L, median (IQR)	1.69 (1.31—2.19)	1.7 (1.35—2.2)	1.64 (1.29—2.16)	0.39
Platelets, N*10^9^/L, median (IQR)	230 (195—278)	253 (210—309)	222 (192—265)	** < 0.01**
LDH increase (> 300 U/L), n. (%)	54 (20.45)	18 (22.78)	36 (19.46)	0.54
C3 reduction (< 90 mg/dL), n. (%)	57 (21.67)	16 (20.78)	41 (22.04)	0.82
C4 reduction (< 10 mg/dL), n. (%)	35 (13.16)	9 (11.39)	26 (13.90)	0.58
C3 or C4 reduction, n. (%)	70 (26.32)	21 (26.58)	49 (26.20)	0.95
Total cholesterol (mg/dL), median (IQR)	191 (168—219)	191 (170—217)	190 (164—219)	0.77
HDL cholesterol (mg/dL), median (IQR)	62 (52—78)	62 (51—78)	62 (52—78)	0.89
LDL cholesterol (mg/dL), median (IQR)	105 (86—125)	107 (81—131)	105 (87—125)	0.48
Triglycerides (mg/dL), median (IQR)	84 (60 −111)	85 (60 −110)	79 (60 −114)	0.78
Fasting glycemia (mg/dL), median (IQR)	86 (79—95)	85 (79—95)	87 (78—94)	0.63
Hypergammaglobulinemia, n. (%)	93 (35.09)	22 (27.85)	71 (38.17)	0.11
Cryoglobulins test positive, n. (%)	26 (9.85)	7 (8.97)	19 (10.22)	0.76
β2-Microglobulin (mg/dL), median (IQR)	0.17 (0—0.25)	0.19 (0.01—0.26)	0.15 (0—0.25)	0.33
IgA level (mg/dL), median (IQR)	227 (155—299)	227 (177—294)	223 (133—301)	0.15
IgG level (mg/dL), median (IQR)	1310 (1005—1741)	1270 (1005—1620)	1368 (1007—1837)	0.58
IgM level (mg/dL), median (IQR)	103 (61—164)	99 (60—193)	103 (65—159)	0.98
ANA positive, *n*. (%)	205 (79.15)	62 (79.49)	143 (79.01)	0.93
Anti-SSA positive *n*. (%)	182 (70.27)	50 (64.1)	132 (72.93)	0.15
Anti-SSB positive, *n*. (%)	97 (37.45)	25 (32.05)	72 (39.78)	0.24
RF positive, *n*. (%)	50 (19.38)	16 (20.51)	34 (18.89)	0.76

*ANA* anti-nuclear antibodies, *CRP* C-reactive protein, *C3* complement component 3, C4, complement component 4, *ESR* erythrocyte sedimentation rate, *FM* fibromyalgia, *HDL* high-density lipoprotein, *Ig* immunoglobulins, *IQR* interquartile range, *LDH* lactate dehydrogenase, *LDL* low-density lipoprotein, *n*., number, *RF* rheumatoid factors, *SjD* Sjögren’s disease, *SSA* Sjögren’s syndrome-related antigen A, *SSB* Sjögren’s syndrome-related antigen B

### Differences in pharmacological management according to fibromyalgia status

Treatment with csDMARD was administered in 85% of patients, while bDMARD were used in only 7% of cases. Although not statistically significant (*p* = 0.088), bDMARD use tended to be more frequent in patients with FM (11.3 vs. 5.4%). Prior CS use was significantly more common in patients with FM (82.5% vs. 66.3%; *p* = 0.008), whereas ongoing CS therapy and median daily doses were similar between groups. Pilocarpine was administered to 40% of patients and more frequently among those with FM (47.5% vs. 37.1%), although the difference did not reach statistical significance (*p* = 0.113).

Symptom-targeted therapies were substantially more frequent in FM patients: antidepressants were used in 40% vs. 2.7%, and muscle relaxants in 73.8% vs. 13.4% (*p* < 0.001, for both) (Table 4).

**Table 4:** Differences in pharmacological management according to fibromyalgia status

Treatments administered	SjD (*n*. 267)	SjD with FM (*n*. 80)	SjD without FM (*n*. 187)	*p*-value
csDMARD, *n*. (%)	226 (84.64)	68 (85.00)	158 (84.49)	0.92
bDMARD, *n*. (%)	19 (7.14)	9 (11.25)	10 (5.38)	0.09
Previous CS, *n*. (%)	190 (71.16)	66 (82.50)	124 (66.31)	** < 0.01**
CS use, *n*. (%)	102 (38.20)	34 (42.50)	68 (36.36)	0.34
PDNeq dose (mg/day), median (IQR)	0 (0—2)	0 (0—5)	0 (0—2)	0.15
Pilocarpine, *n*. (%)	107 (40.23)	38 (47.50)	69 (37.10)	0.11
Antidepressants, *n*. (%)	37 (13.86)	32 (40.00)	5 (2.67)	** < 0.001**
Muscle relaxants, *n*. (%)	84 (31.46)	59 (73.75)	25 (13.37)	** < 0.001**

*bDMARD* biologic disease-modifying antirheumatic drugs, *CS* corticosteroids, *csDMARD* conventional-synthetic disease-modifying antirheumatic drugs, *FM* fibromyalgia, *IQR* interquartile range, *n*. Number, *PDNeq* prednisone equivalent, *SjD* Sjögren’s disease

### Factors associated with fibromyalgia in patients with Sjögren’s disease

In patients with concomitant FM, the univariate logistic regression analysis revealed a significant association with the presence of MADD (OR 3.35, 95% CI 1.52–7.34; *p* = 0.003), CS use (OR 2.40, 95% CI 1.25–4.60; *p* = 0.009), and higher symptom burden as measured by the ESSPRI score (OR 1.52, 95% CI 1.29–1.79; *p* < 0.001). All ESSPRI domains were individually associated with FM, including fatigue (OR 1.55, 95% CI 1.32–1.83; *p* < 0.001), pain (OR 1.34, 95% CI 1.19–1.51; *p* < 0.001), and dryness (OR 1.14, 95% CI 1.02–1.28; *p* = 0.021) (Table 5).

**Table 5:** Factors associated with fibromyalgia in patients with Sjögren’s disease

Logistic regression analysis	Univariate		Multivariate	
Variables	OR (95% CI)	*p*-value	aOR (95% CI)	*p*-value
MADD	3.35 (1.52—7.34)	** < 0.01**	**3.24 (1.13—9.30)**	**0.03**
Arthralgia	1.79 (1.05—3.03)	**0.03**		
Antidepressant use	24.27 (8.97—65.62)	** < 0.001**		
Muscle relaxant use	18.21 (9.48—34.95)	** < 0.001**		
CS use	2.40 (1.25—4.60)	** < 0.01**	**2.76 (1.02–7.48)**	**0.05**
Platelet number	1.01 (1.00—1.01)	** < 0.01**		
ESSPRI	1.52 (1.29—1.79)	** < 0.001**	**1.36 (1.13—1.62)**	** < 0.001**
Pain VAS	1.34 (1.19—1.51)	** < 0.001**		
Fatigue VAS	1.55 (1.32—1.83)	** < 0.001**		
Dryness VAS	1.14 (1.02—1.28)	**0.02**		
Age	0.99 (0.97—1.01)	0.20	0.98 (0.95—1.01)	0.12
Male sex	0.18 (0.02—1.44)	0.11		
Disease duration	1.00 (1.00—1.00)	0.43	1.00 (0.99—1.00)	0.84

*aOR* adjusted odds ration, *CS* corticosteroids, *CI* confidence intervals, *ESSPRI* EULAR sjögren syndrome patient-reported index, *FM* fibromyalgia, *MADD* mixed anxiety–depressive disorder, *OR* odds ratio, *SjD* Sjögren’s disease, *VAS* visual analog scale

After adjustment for pharmacological therapies (CS, antidepressants, muscle relaxants, and pilocarpine), the multivariate analysis revealed both the overall ESSPRI score—and its individual domains—associated with FM. Specifically, ESSPRI score (aOR range: 1.39–1.51; all *p* < 0.001), fatigue VAS (aOR range: 1.40–1.55; all *p* < 0.001), and pain VAS (aOR range: 1.21–1.34; all *p* < 0.001) retained statistical significance across all models. In contrast, the association with dryness was attenuated in adjusted models. While it remained significant after adjusting for CS (aOR 1.14; *p* = 0.028) and pilocarpine (aOR 1.13; *p* = 0.04), it was no longer significant when adjusting for antidepressants (aOR 1.09; *p* = 0.214) or muscle relaxants (aOR 1.08; *p* = 0.262).

In the final multivariate model, adjusted for age and disease duration, three variables retained independent association with FM: higher ESSPRI score (aOR 1.36, 95% CI 1.13–1.62; *p* = 0.001), MADD (aOR 3.24, 95% CI 1.13–9.30; *p* = 0.029), and CS use (aOR 2.76, 95% CI 1.02–7.48; *p* = 0.046) (Table 5).

## Discussion

This multicenter study sheds light on the substantial clinical burden posed by FM in patients with SjD, highlighting a prevalence of 30% and a distinct symptom-driven phenotype. While demographic differences such as age and sex did not reach statistical significance, patients with concomitant FM exhibited a markedly more complex clinical picture, with higher scores across all ESSPRI domains and greater use of symptomatic treatments, including CS and antidepressants.

The coexistence of FM is being increasingly recognized across autoimmune diseases, including RA, SpA, SLE, and systemic sclerosis (SSc) [21, 22]. Several studies reported FM prevalence in SjD ranging from 15% to nearly 50%, depending on cohort size, diagnostic criteria, and study design. The presence of FM was consistently associated with higher symptom burden, particularly fatigue, widespread pain, and depression, as well as elevated patient-reported indices such as ESSPRI, despite more frequent symptomatic treatments [23–25]. Other smaller studies reported similar findings, with FM contributing to increased psychological distress and sleep disturbances, potentially compounding the complexity of clinical assessment [26–28]. While dryness is considered a hallmark of SjD, it is also reported in up to one-third of FM patients, further blurring the clinical boundaries between these conditions [29, 30].

In our cohort, we compared patient-reported (ESSPRI) and physician-assessed (ESSDAI) disease activity between patients with and without FM. Interestingly, while ESSDAI scores did not differ significantly, ESSPRI was markedly higher in patients with concomitant FM. All three ESSPRI domains were significantly more elevated, reflecting a distinct symptom amplification pattern in FM patients. This dissociation between objective disease activity and subjective symptom burden underscores the limitations of relying solely on patient-reported indices in the assessment of SjD, especially in those with overlapping FM, where pain and fatigue may reflect central sensitization rather than inflammatory activity. The discrepancy between clinical activity and patient-reported symptoms in SjD-FM patients suggests that ESSPRI may overestimate the inflammatory burden, potentially reflecting mechanisms of central sensitization. This should be carefully evaluated during clinical assessment, especially if treatment with CS or DMARD is being considered, as this was shown to be largely ineffective.

Among comorbidities, only MADD was more frequent in SjD patients with concomitant FM. Regarding laboratory data, the platelet counts were higher in patients with concomitant FM, but this difference was not considered clinically relevant, with similar frequency of thrombocytosis between groups. Clinical involvement was otherwise largely comparable between groups, except for arthralgias, which were more commonly reported in FM subgroup, probably reflecting heightened pain sensitivity rather than true joint inflammation. Interestingly, while a Dutch study suggested that SjD patients with widespread pain may represent a benign subset, characterized by lower prevalence of anti-SSB antibodies and less extra-glandular involvement, such as polyneuropathy [31], our data did not confirm this hypothesis. The prevalence of extra-glandular manifestations, including polyneuropathy, and the immunological profile (anti-SSA/SSB antibodies) were comparable between patients with and without FM.

In our cohort, patients with concomitant FM showed higher ESSPRI scores across all domains, highlighting how FM amplifies the perceived symptom burden in SjD. Despite its known impact on the quality of life, the role of FM in modulating treatment response in SjD remains poorly defined [32]. Data from RA and other rheumatic diseases suggest that FM may blunt the perceived therapeutic benefits. In SjD, DMARD often fail to improve ESSPRI domains, especially in the presence of FM, underscoring the need to account for FM when evaluating treatment effectiveness [33–35]. Interestingly, in our cohort treatment with bDMARD was slightly more frequent among patients with FM. Although this difference did not reach statistical significance, it supports the hypothesis that SjD patients with concomitant FM tend to report greater symptom severity, which may influence physicians toward more intensive treatment strategies. In our cohort, patients with FM more often received CS, muscle relaxants, and antidepressants. After adjustment for treatments, higher ESSPRI scores, particularly in the pain and fatigue domains, remained associated with FM. These findings suggest that FM-related symptoms may persist irrespective of pharmacological interventions. Previous studies confirm that FM is associated with higher ESSPRI but not ESSDAI scores in SjD, with depression emerging as a key driver of fatigue [36, 37]. Our data align with this trend but also suggest that increased dryness scores in FM patients may be partially influenced using muscle relaxants and antidepressants.

Although the pathogenesis of both SjD and FM remains incompletely understood, their frequent clinical overlap has prompted speculations about shared immunological mechanisms. In a cohort study of FM patients with sicca symptoms, one-third tested positive for tissue-specific autoantibodies associated with early-stage SjD, suggesting a possible autoimmune component in FM [38]. Other novel antibodies, such as anti-aquaporin-5, have been implicated in SjD and may influence its clinical expression [39]. Finally, a pilot study using salivary gland ultrasonography in FM patients revealed a high prevalence of sicca symptoms and structural changes in these subjects, suggesting that a subset of FM patients may harbor subclinical or misdiagnosed SjD [40].

This multicenter real-life study is one of the largest to describe the prevalence and clinical profile of FM in Italian patients with SjD, integrating both subjective and objective disease activity measures and adjusting for treatment effects. Main limitations include its retrospective design, potential reporting bias, and lack of data on topical therapies and lifestyle factors. Moreover, longitudinal data regarding treatment response were not available, dampening the strength of our results. Despite this, the study provides clinically relevant insights into the SjD–FM overlap, supporting the need for prospective studies and biomarkers to better differentiate inflammatory and non-inflammatory symptoms.

## Conclusions

The presence of FM should be carefully considered when interpreting disease activity in patients with SjD. In our study, ESSDAI scores were similar regardless of FM status, while ESSPRI was significantly higher in patients with FM, with all its components contributing to this difference.

These findings highlight the need for caution when using patient-reported indices such as ESSPRI in SjD patients with FM, as they may reflect symptom amplification rather than true inflammatory activity.

## Supplementary Information

Below is the link to the electronic supplementary material.

Supplementary file1 (DOCX 16 KB)

## Data Availability

The data that support the findings of this study are available from the corresponding author upon reasonable request.
