# Supplementary material for: Sjögren’s disease and concomitant fibromyalgia: clinical profile and implications for disease activity assessment
Source: Intern Emerg Med. 2025 Nov 11;21(2):477–85. doi: 10.1007/s11739-025-04193-x (PMC13061780; doi:10.1007/s11739-025-04193-x)
Supplement: Supplementary file 1 — Supplementary file1 (DOCX 16 KB) [file 11739_2025_4193_MOESM1_ESM.docx]

**Supplementary table 1.** Variables included in the multivariate regression analysis before backward selection**.**

| **Variables** | **OR** | **95% CI** | **p-value** |
| --- | --- | --- | --- |
| **Age** | 0.99 | 0.97 - 1.01 | 0.20 |
| **Male sex** | 0.18 | 0.02 - 1.44 | 0.11 |
| **CS use** | 2.40 | 1.25 - 4.60 | **<0.01** |
| **CS dose** | 1.09 | 0.98 - 1.22 | 0.11 |
| **bDMARD use** | 2.23 | 0.87 - 5.72 | 0.09 |
| **Antidepressants use** | 24.27 | 8.97 - 65.62 | **<0.001** |
| **Muscle relaxants use** | 18.21 | 9.48 - 34.95 | **<0.001** |
| **Pilocarpine use** | 1.53 | 0.90 - 2.61 | 0.11 |
| **MADD** | 3.35 | 1.52 - 7.34 | **<0.01** |
| **Arthralgia** | 1.79 | 1.05 - 3.03 | **0.03** |
| **Increased CRP** | 1.70 | 0.82 - 3.55 | 0.16 |
| **Hypergammaglobulinemia** | 0.63 | 0.35 - 1.11 | 0.11 |
| **Platelets number** | 1.01 | 1.00-1.01 | **<0.01** |
| **C3 level** | 1.10 | 1.00 - 1.02 | 0.14 |
| **SSA positive** | 0.66 | 0.38 - 1.17 | 0.15 |
| **Predicted FVC** | 1.02 | 1.00 - 1.01 | 0.10 |
| **ESSPRI** | 1.52 | 1.29 - 1.79 | **<0.001** |
| **Pain VAS** | 1.34 | 1.19 - 1.51 | **<0.001** |
| **Fatigue VAS** | 1.55 | 1.32 - 1.83 | **<0.001** |
| **Dryness VAS** | 1.14 | 1.02 - 1.28 | **0.02** |

Abbreviations: bDMARD, biologic disease-modifying antirheumatic drugs; CS, corticosteroids; CI, confidence intervals; CRP, C-reactive protein; C3, complement component 3; ESSPRI, EULAR Sjögren Syndrome patient reported index; FM, Fibromyalgia; FVC, forced vital capacity; MADD, Mixed anxiety-depressive disorder; OR, odds ratio; SjD, Sjögren’s disease; SSA, Sjögren’s syndrome-related antigen A; VAS, visual analogue scale.
